# Zoonotic Viral Diseases of Equines and Their Impact on Human and Animal Health

**DOI:** 10.2174/1874357901812010080

**Published:** 2018-08-31

**Authors:** Balvinder Kumar, Anju Manuja, BR Gulati, Nitin Virmani, B.N. Tripathi

**Affiliations:** ICAR-National Research Centre on Equines, Hisar-125001, India

**Keywords:** Zoonotic diseases, Equine viral diseases, Human health, Animal health, Viral diseases, Pandemics

## Abstract

**Introduction:**

Zoonotic diseases are the infectious diseases that can be transmitted to human beings and vice versa from animals either directly or indirectly. These diseases can be caused by a range of organisms including bacteria, parasites, viruses and fungi. Viral diseases are highly infectious and capable of causing pandemics as evidenced by outbreaks of diseases like Ebola, Middle East Respiratory Syndrome, West Nile, SARS-Corona, Nipah, Hendra, Avian influenza and Swine influenza.

**Expalantion:**

Many viruses affecting equines are also important human pathogens. Diseases like Eastern equine encephalitis (EEE), Western equine encephalitis (WEE), and Venezuelan-equine encephalitis (VEE) are highly infectious and can be disseminated as aerosols. A large number of horses and human cases of VEE with fatal encephalitis have continuously occurred in Venezuela and Colombia. Vesicular stomatitis (VS) is prevalent in horses in North America and has zoonotic potential causing encephalitis in children. Hendra virus (HeV) causes respiratory and neurological disease and death in man and horses. Since its first outbreak in 1994, 53 disease incidents *have been reported in* Australia. West Nile fever has spread to many newer territories across continents during recent years.

It has been described in Africa, Europe, South Asia, Oceania and North America. Japanese encephalitis has expanded horizons from Asia to western Pacific region including the eastern Indonesian archipelago, Papua New Guinea and Australia. Rabies is rare in horses but still a public health concern being a fatal disease. Equine influenza is historically not known to affect humans but many scientists have mixed opinions. Equine viral diseases of zoonotic importance and their impact on animal and human health have been elaborated in this article.

**Conclusion:**

Equine viral diseases though restricted to certain geographical areas have huge impact on equine and human health. Diseases like West Nile fever, Hendra, VS, VEE, EEE, JE, Rabies have the potential for spread and ability to cause disease in human. Equine influenza is historically not known to affect humans but some experimental and observational evidence show that H3N8 influenza virus has infected man. Despite our pursuit of understanding the complexity of the vector-host-pathogen mediating disease transmission, it is not possible to make generalized predictions concerning the degree of impact of disease emergence. A targeted, multidisciplinary effort is required to understand the risk factors for zoonosis and apply the interventions necessary to control it.

## 1 INTRODUCTION

Zoonotic diseases are the infectious diseases that can be transmitted to human beings from animals and vice versa either directly through contact or indirectly through contaminated inanimate objects, intermediate hosts and bites of insect vectors *etc* [1]. Out of the 1407 pathogens affecting human beings, 816 (58%) are of animal origin and approximately 73% of emerging human pathogens are zoonotic in nature [2].The pathogens continue to spread due to increased and faster movement of animals to newer locations, expanding international trade, increasing urbanization, environmental changes, increasing numbers of immune-compromised patients and many more associated factors [3]. Zoonotic viral diseases like Ebola, Middle East Respiratory Syndrome, West Nile, SARS-Corona, Nipah, Hendra, Avian influenza and Swine influenza are the examples of diseases which have threatened health and economies around the world. Japanese encephalitis virus (JEV) has spread throughout Asia and Australia and has also been reported in non-endemic countries like the United States in travel-associated cases [4, 5]. Increased movement of equines for trade, sports, breeding or other purposes has also enhanced the possibility of the spread of equine diseases to newer territories. Many equine viral diseases like Eastern equine encephalitis (EEE), Western equine encephalitis (WEE), and Venezuelan-equine encephalitis (VEE) can be also be disseminated through aerosols and are highly infectious [6-8]. Vesicular stomatitis (VS) is prevalent in many countries of the world and has zoonotic potential [9]. Infectious disease caused by Hendra virus has shown its impact by the death of horses and people in Australia [10].West Nile fever has spread to many new territories during recent years [11]. Rabies is relatively rare in equines but still a public health concern [12-15]. Rabies cases have been reported in mules [16] and donkeys [17]. Middle East Respiratory Syndrome-Corona Virus (MERS-CoV) is another emerging zoonotic virus. MERS-CoV needs dipeptidyl-peptidase-4 (DPP-4) receptors on host cells for infection. Molecular studies have revealed similarity between human and equine dipeptidyl-peptidase-4 (DPP-4) receptors for viral spike proteins of MERS-CoV indicating possible susceptibility of horses to this novel virus [18-20]. Equine influenza is historically not known to affect humans but many scientists have mixed opinions. Equine influenza H3N8 viruses have been reported to infect man occasionally.

In this article, an overview of viral diseases common to equines and human beings along with a focus on their impact is presented. The information will be useful to increase awareness about these diseases and guide devising effective strategies for the prevention and control of these infections.

### 1.1 West Nile Viral Encephalitis

West Nile virus (WNV) is a zoonotic *Flavivirus* belonging to the family *Flaviviridae* [21]. The virus is transmitted by mosquitoes and causes fatal encephalitis in human [22-27], equines [28-30] and birds [31-34].

WNV was first isolated and identified in 1937 from a woman presented with mild febrile illness in the Nile district of Uganda [35]. It has been described in Africa, Europe, South Asia, Oceania and North America [36-38]. Countries with incidence/serological evidence are presented in Fig. (**1**). In North America, more than 1.8 million people have been infected, with over 12,852 reported cases of encephalitis or meningitis and 1,308 deaths from 1999 to 2010 [39]. The mortality rate in human varies from 3-15% and can reach up to 50% in clinically affected horses [40]. WNV in India has been confirmed by seroprevalence and by virus isolation on different occasions from mosquitoes [41-43] bat [44] and man [42, 45-47]. WNV infection has also been reported in animals and birds [48-52].

Horses and human are the main hosts. Animals other than horses may be susceptible to WNV, but rarely become ill. Antibodies have been found in serum samples from bats, horse, dogs, cats, racoons, opossums, squirrels, domestic rabbits, eastern striped skunks, cows, sheep, deer and pigs [53-60]. The virus is transmitted to humans by mosquitoes. About 20% of the infected people develop fever with other symptoms. Fatal, neurologic illness occurs in less than 1% of infected people [61].

WNV is amplified by continuous transmission cycles between mosquitoes and birds. Generally *Culex* mosquitoes are the vectors and passerine birds are the vertebrate reservoirs in enzootic transmission cycles. The virus is carried in the salivary glands of infected mosquitoes and transmitted to susceptible birds during blood-sucking. Competent bird reservoirs sustain an infectious viraemia for 1 to 4 days subsequent to exposure, and then develop life-long immunity. Horses, human and most other mammals rarely develop the infectious levels of viraemia and are dead-end hosts. Few cases in human have been spread through blood transfusions, organ transplants, breast feeding and during pregnancy [62]. The ticks observed to be infected naturally include *Ornithodoros maritimus, Argas hermanni* and *Hyalomma marginatum* and the virus has also been isolated from other species of hard ticks in Africa, Europe and Asia [36, 63, 64]. Swallow bugs (*Oeciacus hirundinis*) have been implicated as vectors in Austria [36]. In most of the horses bitten by carrier mosquitoes, there is no disease. Approximately 33% of the infected horses develop severe disease and die or are affected severely [65-67]. The time between the bite of an infected mosquito and appearance of clinical signs ranges from 3 to 14 days. The symptoms in horses may vary from none to trembling, skin twitching and ataxia [68]. There can be sleepiness, dullness, listlessness, facial paralysis, difficulty in urination and defecation, and inability to rise. In some horses, there can be mild fever, blindness, seizures, and other signs.

There is no effective treatment for clinical WNV infection in humans, horses or any other animal. Vaccines are available for control of WNV in horses in the USA [68-70]. Vaccination of horses protects valuable animals from a potentially fatal disease, but trade and competition practices make this undesirable as some countries use positive antibody tests and impose import restrictions. WNV encephalitis is so rare in human that vaccine development may not be feasible economically.

### 1.2 Hendra

Hendra virus (HeV) is a rare, emerging zoonotic virus. It causes respiratory and neurological disease and death in man and horses [71]. HeV is a member of genus *Henipavirus* of family *Paramyxoviridae*, order *Mononegavirales* containing two members, Hendra and Nipah viruses [21].The potential for rapid spread and ability to cause disease in man have made it a public health concern. The shift from respiratory to neurological symptoms has further raised the concerns [72]. HeV was first identified in 1994 during the first recorded outbreak of the disease in Australia [73]. Up to 2016, 53 disease incidents affecting more than 70 horses and 7 human beings have been reported mainly confined to the east coast of Australia [74].

HeV has been documented to infect horses, humans, dogs and flying foxes naturally [75-77]. Experimentally cats, pigs, hamsters, ferrets, African green monkeys and guinea pigs have been documented to exhibit symptoms when experimentally infected [78-83]. Mice are susceptible to HeV infection when exposed via the intranasal route, but resist infection when challenged by a parenteral route [84]. African fruit bats of the genus *Eidolon*, family *Pteropodidae*, have been found serologically positive for HeV antibodies indicating it’s prevalence in Africa [72]. The HeV can be transmitted through body fluids, tissues or excretions of HeV infected horses. No human-to-human transmission has been reported till date [85]. Fruit bats of the family *Pteropodidae,* particularly the species belonging to the *Pteropus* are the natural hosts for HeV. There are no visible disease symptoms in fruit bats. The transmission route is likely through contamination of pasture or feed by infected uterine fluids or fetal tissues from bats [86, 87]. Horses can be infected after the exposure to HeV present in the urine of infected bats. No evidence of transmission to humans from bats has been reported [77]. Human infections range from mild influenza-like illness to fatal respiratory or neurological disease. Infected people develop fever, headaches, myalgia, sore throat and a dry cough [88]. The viral genetic material has been recovered from nasal swabs from horses even after 2 days of experimental infection with an increase in viral loads after replication in upper respiratory tract or nasopharynx followed by viremia during clinical manifestations. This suggests the risk of transmission to human beings during preclinical stages in horse [79].

Infections in horses range from asymptomatic infection to fatal respiratory and neurological syndromes. For fatal cases, the course of illness takes an average of two days. Symptoms of infection in horses are not distinctly different from other respiratory and neurological illnesses of horses. The outbreaks in horses occur one to two weeks before illness in humans which could trigger prevention measures to prevent associated outbreaks in humans. The incubation period in horses varies between 4 and 16 days. The mortality rate in horses is about 75% [71].

There are currently no drugs available to treat HeV infection. Symptomatic treatment with supportive care is the main approach to managing the infection in people. HeV vaccine for horses, EquivacHeV (Zoetis, Parkville, VIC., Australia) has been developed [71]. The vaccine proved very effective at preventing HeV infection in experiments. The vaccine effectively checks the transmission of the disease from flying foxes to horses.

### 1.3 Vesicular Stomatitis

Vesicular stomatitis is a viral disease which primarily affects cattle, horses, and swine. It occurs in enzootic and epizootic forms in the tropical and subtropical areas [89]. The disease is rarely life-threatening but can have a significant financial impact on the horse industry. Vesicular stomatitis virus (VSV) is the prototype of the genus *Vesiculovirus* in family *Rhabdoviridae* [21]. The virus has two serologically distinct serotypes, VSV-New Jersey (NJ) and VSV-Indiana (IND). The neutralizing antibodies generated by these two serotypes are not cross-reactive. The IND serogroup has three subtypes IND-1 (classical IND) IND-2 (cocal virus) and IND-3 (alagoas virus) The virus is endemic in South America, Central America, Southern Mexico, Venezuela, Colombia, Ecuador and Peru but the disease has been reported in South Africa in 1886 and 1897 and France in years 1915 and 1917 [90].

The disease has been reported across continents in Belize, Bolivia, Brazil, Colombia, Costa Rica, Ecuador, El Salvador, Guatemala, Honduras, Mexico, Nicaragua, Pakistan, Panama, Peru, USA and Venezuela [91, 92]. Outbreaks historically occurred in all regions of the USA but have been limited to western states in 1995, 1997, 1998, 2004, 2005, 2006, 2009, 2010, and 2012 [93, 94]. While VS has been reported in horses at about 800 premises in eight states [95]. VSV spread to Europe during the First World War and periodically appears in South Africa. The Chandipura virus, a *Vesiculovirus* caused encephalitis outbreaks in different states of India leading to mortalities in children [96]. Isfahan another virus in this genus is endemic in Iran [89, 97]. The countries with incidence/serological evidence of vesicular stomatitis are presented in Fig. (**2**).

Clinical disease has been observed in cattle, horses, pigs and camels whereas sheep, goats and llamas tend to be resistant. White-tailed deer and numerous species of small mammals in the tropics are considered as wild hosts. Many species, including cervids, nonhuman primates, rodents, birds, dogs, antelope, and bats have shown serological evidence of infection [98-100]. Experimentally different animals like mice, rats, guinea-pig, deer, raccoons, bobcats, and monkeys can be infected.

The virus is zoonotic and causes flu-like symptoms characterized by fever, chills, nausea, vomiting, headache, retrobulbar pain, myalgia, sub-sternal pain, malaise, pharyngitis, conjunctivitis, and lymphadenitis in humans. Vesicular lesions may be present in the pharynx, buccal mucosa, or tongue [101-106]. Encephalitis is rare but may occur in children [107, 108].

The transmission is more likely by trans-cutaneous or transmucosal route. The virus can be transmitted through direct contact with infected animals having lesions of the disease or by blood-feeding insects. In endemic areas, *Lutzomyia* sp. (sand fly) is proven biologic vectors. Black flies (*Simulidae*) are the most likely biologic insect vector in USA. Other insects may also act as mechanical vectors. Saliva, exudates and epithelium from open vesicles are sources of virus. Plants and soil are also suspected as the source of virus.

Horses of all ages appear equally susceptible [109] but lesions do not appear in all susceptible horses [110]. The lesions of the disease resemble foot-and-mouth disease in cattle and the other viral vesicular diseases in pigs. The horses are resistant to foot and mouth disease and susceptible to VS. VSV is the only viral vesicular disease of livestock that infects horses [111]. VSV is also the most important of these four viruses as a zoonotic agent for humans. When vesicular stomatitis occurs in horses, blanched raised or broken vesicles or blister-like lesions develop on the tongue, mouth lining, nose and lips [112]. In some cases, lesions also develop on the udder or sheath or the coronary bands of horses. Animals may become anorectic, lethargic and have pyrexia. One of the most obvious clinical signs is drooling of saliva or frothing at the mouth. The rupture of the blisters creates painful ulcers in the mouth. The surface of the tongue may slough. Excessive salivation is often mistaken as a dental problem or colic [113]. There may be weight loss due to mouth ulcers as animal finds it too painful to eat. The lesions around the coronary band may cause lameness and laminitis. In severe cases, the lesions on the coronary band may cause the hoof to slough. Animals usually recover completely within two weeks. Morbidity rates vary between 5 and 70% but mortality is rare. Vesicular stomatitis like disease disabled 4000 horses during the Civil War in 1862. Major epidemics in the US occurred in 1889, 1906, 1916, 1926, 1937, 1949, 1963, 1982, and 1995, with minor outbreaks during many other years [114]. No specific treatment is available for the disease. Anti-inflammatory medications as supportive care help to minimize swelling and pain. Dressing the lesions with mild antiseptics may help avoid secondary bacterial infections. If fever, swelling, inflammation or pus develops around the sores, treatment with antibiotics may be required. The animals should be quarantined at least for 21 days after recovery of the last case before moving to other places. Vaccines for livestock are available in some Latin American countries.

### 1.4 Eastern Equine Encephalitis

Eastern equine encephalitis (EEE) commonly called triple E or, sleeping sickness is a rare but serious viral disease affecting horses and man. The disease is transmitted through mosquitoes and man and horses are dead-end hosts [115].

EEEV belongs to the genus *Alphavirus* of the family *Togaviridae*. It is closely related to Venezuelan equine encephalitis (VEE) virus and Western equine encephalitis (WEE) virus [116]. This virus has North American and South American variants. The North American variant is more pathogenic. EEE is capable of infecting a wide range of animals including mammals, birds, reptiles and amphibians [117]. The virus has been reported to cause disease in poultry, game birds and ratites. The disease has also been reported to occur in cattle, sheep, pigs, deer, and dogs though sporadically. The disease is present in North, Central and South America and the Caribbean. EEE was first recognized in the USA in 1831 from an outbreak where 75 horses died of encephalitic illness and EEE virus (EEEV) was first isolated from infection horse brain in 1933 [118]. The serological evidence and outbreaks of the disease have also been reported from horses in Canada and Brazil [119, 120]. Countries with incidence/serological evidence are presented in Fig. (**3**). EEEV infection in horses is often fatal. The human cases were identified first time in 1938 in the north-eastern United States. Thirty children died of encephalitis in this outbreak. The fatality rate in humans was 35%. The outbreaks of the disease also occurred in horses simultaneously in the same regions. A total of 19 human cases of the disease were reported in children between 1970-2010 in Massachusetts and New Hampshire [121]. As per the CDC reports 220 confirmed human cases of the disease occurred in the U.S. from 1964 to 2004 [122]. In 2007, a citizen of Livingston, West Lothian, Scotland became the first European victim of this disease after infected with EEEV from New Hampshire. EEE has been diagnosed in Canada, the United States of America (USA), the Caribbean Islands and Mexico [122, 123]. Eighteen cases of Eastern equine encephalomyelitis occurred in six Brazilian states between 2005 and 2009 [120].

Alternate infection of birds and mosquitoes maintains these viruses in nature. *Culiseta melanura* and *Cs. morsitans* species are primarily involved. Transmission of EEEV to mammals occurs via other mosquitoes which are primarily mammalian feeders and called as bridge vectors [124]. Infected mammals do not circulate enough viruses in their blood to infect additional mosquitoes. The virus is introduced by mosquitoes, but feather picking and cannibalism also contribute towards the transmission of the disease within the flocks [125]. Most people bitten by an infected mosquito do not develop any symptoms. The symptoms generally appear 3 to 10 days after the bite of an infected mosquito. The clinically affected patients may have pyrexia, muscle pains, headache, photophobia, and seizures. EEEV is one of the potential biological weapons. The disease in horses is characterized by fever, anorexia, and severe depression. Symptoms appear one to three weeks post-infection, and begin with a fever that may be as high as 106ºF. The fever usually lasts for 24–48 hours. In severe cases, the disease in horses progresses to hyper-excitability, blindness, ataxia, severe mental depression, recumbency, convulsions, and death [126]. The nervous symptoms may appear due to brain lesions. This may be followed by paralysis, causing the horse to have difficulty raising its head. The horses usually suffer complete paralysis and die two to four days after symptoms appear. Mortality rates among horses range from 70 to 90% [127].

There is no cure for EEE. Severe illnesses are treated by supportive therapy consisting of corticosteroids, anticonvulsants, intravenous fluids, tracheal intubation, and antipyretics. Vaccines containing killed virus are used for prevention of the disease. These vaccinations are usually given as combination vaccines, most commonly with WEE, VEE, and tetanus. Elimination of mosquito breeding sites and use of insect repellents may help in control of the disease.

### 1.5 Venezuelan Equine Encephalitis

Venezuelan equine encephalitis (VEE) is an arbovirus infection transmitted by mosquitoes. VEE viruses (VEEV) are classified in the genus *Alphavirus,* family *Togaviridae*. The VEE virus complex is composed of six subtypes (I–VI); Subtype I includes five antigenic variants (AB–F), of which variants 1-AB and 1-C are associated with epizootics in equines and concurrent epidemics in humans [128]. The epizootic variants 1-AB and 1-C are thought to originate from mutations of the enzootic 1-D serotype [129]. The enzootic strains are 1-D, 1-E and 1-F of subtype I, subtype II, four antigenic variants (A–D) of subtype III, and subtypes IV–VI. The enzootic viruses do not produce clinical encephalomyelitis in the equines normally. Enzootic VEE strains have been identified as Everglades (subtype II) in the Florida, variant I-E in Central American countries and Mexico, variants I-D and I-E in Panama, variant I-D in Venezuela, Colombia, variants 1-D, III-C, and III-D in Peru, variant III-B and subtype V in French Guiana, variant I-D in Ecuador, variant III-A in Suriname and Trinidad, variants I-F and III-A and subtype IV in Brazil and subtype VI in Argentina. In an atypical ecological niche, variant III-B has been isolated in the USA (Colorado and South Dakota) in an unusual association with birds [128]. Countries with incidence/serological evidence are presented in Fig. (**4**).

The primary vectors for the bird or rodent-mosquito life cycle are members of the *Melanoconion* subgenus (*Culex cedecci*). Epizootic VEEV strains (I-AB and I- C) are transmitted by several mosquito vectors (e.g., *Aedes* and *Psorophora* spp.) to equids [130].

Infections with VEE virus (VEEV) may present, in both humans and horses, as either encephalitic disease or as simply a febrile disease without profound neurologic signs. Horses may die after a very acute course, even without any neurologic signs, but mortality in humans is generally low. Horses are not dead-end hosts for VEEV epizootic strains like they are for EEEV and WEEV. Horses are, in fact, the key reservoir species for the epizootic strains of VEEV that cause clinical disease in both horses and humans [128].

Epizootic subtypes highly pathogenic to equines, can spread rapidly through large populations. Equines are the primary animal species and serve as amplifying hosts for epizootic VEE virus strains. Blood-sucking insects feed on infected horses, pick up this virus and transmit to other animals or humans. Animal like cattle, swine, and dogs, can become infected, but they neither show the signs of the disease nor contribute to spread [131]. Aerosol transmission has been reported in human from laboratory accidents [128, 132]. Infections with both epizootic and enzootic variants are infectious to human beings and can occur in laboratory workers. The workers handling infectious VEE viruses or their antigens should take preventive measures including use of containment facilities and vaccination.

VEE can cause disease in equines including horses, mules, donkeys and zebras. Cattle, swine, chickens and dogs have been shown to seroconvert after epizootics; mortality has been observed in domesticated rabbits, dogs, goats and sheep. Humans also can contract this disease. Epidemics of VEE involving tens of thousands of humans have been reported. The mortality rates in equines during epizootics have been 19-83% while 4-14% in human beings associated with neurological disease [129]

It usually causes influenza like symptoms in adults, but in children and horses it can cause severe encephalitis. Equines may suddenly die or exhibit progressive central nervous system disorders. Infections with VEEV may present, in both humans and horses, as either encephalitic disease or as simply a febrile disease without profound neurologic signs. Horses may die after a very acute course, even without any neurologic signs, but mortality in humans is generally low. Young and immune compromised horses are most likely to develop clinical signs [131]. It causes only low morbidity and mortality in man but high morbidity and mortality in animals.

The epizootic VEE was initially limited to northern and western South America in Venezuela, Colombia, Ecuador, Peru and Trinidad, but the epizootics have been reported in years from 1969 to 1972 in Guatemala, Nicaragua, El Salvador, Honduras, Costa Rica, Belize, Mexico, and the United States of America due to variant 1-AB. Epizootics caused by I-AB or I-C virus have not occurred in North America and Mexico after 1972. Equine and human epizootic VEE viruses were subtype 1-C from Venezuela in 1993, 1995 and 1996 and Colombia in 1995 [128]. In 1960 over 200,000 human cases and more than 100,000 equine deaths were estimated in Central Colombia [133]. Countless cases in horses and 75,000-100,000 human cases with more than 300 fatal encephalitis cases occurred in Venezuela and Colombia in 1995 [134]. Equine disease associated with VEEV-IE occurred in Mexico and human cases of VEEV ID-associated disease occurred in Peru from 1993 to 95 [129]. Subtype II has been isolated from humans and mosquitoes from Florida; subtype III has been isolated from the Rocky Mountains and northern plains states. Sylvatic VEE viruses are endemic in North, Central, and South America in swampy environments with persistent fresh or brackish water. Epizootics have been associated with a mutation to a subtype I (A, B, C, and possibly E), a change in mammalian pathogenesis, and change to several bridge vectors.

Treatment of viral encephalitis is supportive, as there are no specific antiviral therapies. The two VEE vaccines, a modified-live vaccine (TC-83) and an inactivated adjuvant vaccine, have been used in field. Horses were vaccinated with TC-83 vaccine during outbreak in Mexico and Texas in 1971 as equine vaccine was not available but it is still in use for humans working with VEE. Formalin-inactivated virulent VEE virus vaccines are not recommended for use in equids due to risk of residual virulence [128].

### 1.6 Western Equine Encephalitis

Western equine encephalitis (WEE) is an uncommon viral illness of horses and human. WEE virus (WEEV) is an *Alphavirus* of the family *Togaviridae* which is maintained between birds and mosquitoes, occasionally causing disease in humans and equids [135, 136]. This is an arbovirus transmitted by mosquitoes of the genera *Culex* and *Culiseta*. It is a recombinant between Sindbis and Eastern equine encephalitis like viruses [137-139]. It has also been reported to cause disease in poultry, game birds and ratites [115]. WEEV is normally maintained between *Culex tarsalis* mosquitoes and birds. WEE has several subtypes consisting Sindbis, Aura, Ft. Morgan and Y 62–33. WEEV previously isolated in the south and eastern USA has been shown to belong to the HJ virus serogroup.

Horses and humans are often referred to as “dead-end” hosts as the virus does not build to high enough levels in blood to infect other mosquitoes [122]. Most people infected with WEE virus will have either no symptoms or a very mild illness. A small percentage of people, especially infants and elderly people to a lesser extent, may develop encephalitis. Approximately 5-15% of these encephalitis cases are fatal, and about 50% of surviving infants will have permanent brain damage [139].

Geographically, WEEV exists throughout uine deaths were estimated in central America and northern portions of South America, Mexico and Canada. In the US, WEEV exist in the western two third of the country. Outbreaks of the disease have been recorded since 1847. In 1930 about 6000 horses and mules were infected leading to about 50% mortalities in California. The largest epidemic was recorded in 1937 and 1938 in USA and Canada. In 1938 outbreak an estimated 264000 equids were infected with a morbidity of 21.4% [140]. In the USA, WEE is seen primarily in provinces west of the Mississippi River. During 1941, there was an outbreak of WEE in several states of US and Canada causing 300,000 cases of encephalitis in mules and horses and 3336 cases in humans [141]. The 1970s saw 209 human cases; 87 were reported during the 1980s, only 4 cases during the 1990s, and no cases have been reported in the USA or Canada since 1998 [142]. The last documented human case in North America occurred in 1994, and the virus has not been detected in mosquito pools since 2008 [143]. In human, WEEV infections tend to be asymptomatic or cause mild disease after a short incubation period of 2–7 days with nonspecific symptoms, *e.g*., sudden onset of fever, headache, nausea, vomiting, anorexia and malaise [139]. In some cases, additional symptoms of altered mental status, weakness and signs of meningeal irritation may be observed. In a minority of infected individuals, encephalitis or encephalomyelitis occurs and may lead to neck stiffness, confusion, tonic-clonic seizures, somnolence, coma and death. WEEV is considered as agent that the US researched as potential biological weapons before the nation suspended its biological weapons program.

In horses, infections with WEEV begin with fever, inappetence and lethargy, progressing to various degrees of excitability and then drowsiness, ultimately leading to paresis, seizures and coma in 5-10 day course of the disease [144]. The WEEV mortality rate in horses is higher than humans. Mortality of horses showing clinical signs of WEE is 20–50%. These symptomatic horses either progress to recumbency or die from WEE infections.

There is no treatment for WEE other than supportive care. Formalin-inactivated whole viral vaccines for EEE, WEE, and VEE are commercially available in mono-, bi-, or trivalent form. Previously non vaccinated adult horses require booster. For adult horses in temperate climates, an annual vaccine within 4 wk of the start of the arbovirus season is recommended. However, for horses that travel between areas affected by the virus, 2 or even 3 times vaccination in a year is recommended. Mares should be vaccinated 3–4 wk before foaling to induce colostral antibody.

### 1.7 Japanese Encephalitis

Japanese encephalitis (JE) is caused by JE virus belonging to genus *Flavivirus* of the family *Flaviviridae* [21]. There is only one serotype of JEV, but at least five genotypes based on phylogenetic analysis of envelop (E) gene sequences [145]. The virus is widespread in eastern, south-eastern and southern Asian countries and has spread to the western Pacific region including the eastern Indonesian archipelago, Papua New Guinea and Northern Australia and Pakistan [146, 147]. It is most common in areas under paddy cultivation with pig rearing in Vietnam [148, 149]. JE virus circulates throughout the year in tropical areas of Asia amongst birds, swine and mosquitoes. Approximately, 3 billion people live in JEV-endemic countries in Asia where 68,000 cases are reported annually [148, 150, 151]. Horses are the primarily affected domestic animals. The disease also affects humans and is a primary public health concern in Asia. The birds of the family *Ardeidae* (herons and egrets) are natural maintenance reservoir and generate high viraemias upon infection without clinical disease. The disease is transmitted principally by *Culex* spp. (*Culex tritaeniorhynchus)* as it has a wide host range including birds, horses, swine and humans. Pigs act as important amplifiers of the virus producing high viraemias [152, 153]. Other sub-clinically infected animals include cattle, sheep, goats, dogs, cats, chickens, ducks, wild mammals, reptiles and amphibians but generally not contribute to spread of the disease. Horses and humans are the dead-end hosts, in which JEV causes acute encephalitis [154].

Sporadic clinical cases of JE in horses have been reported in various countries including Japan [155], Hong Kong [156], Taiwan [157], and India [158]. In addition, JE sero-positivity among equines has been reported in Nepal, Korea, Indonesia, and India [158-167]. Countries with incidence/serological evidence are presented in Fig. (**5**). In horses three syndromic manifestations have been described. Transitory type syndrome, lethargic type syndrome and hyper-excitability type syndrome [168]. The symptoms may vary from moderate to high fever (41°C or higher) accompanied by profuse sweating and muscle tremors, aimless wandering, behavioural changes manifested by aggression, loss of vision, collapse, coma and death and neurologic sequelae may result. Morbidity rates reported from field cases vary from less than 1 to 1.4%. Mortality rate in outbreaks can vary from 5 to 15% but can reach 30–40% in more severe epizootics [168].

In human beings, most infections are asymptomatic but can manifest as severe encephalitis, and with neurological sequelae in survivors. Japanese encephalitis tends to be a childhood disease in endemic areas but all ages can be affected in a naive population. As per the estimates 68000 clinical cases occur globally each year, with approximately 13600 to 20400 deaths [169]. The first case of Japanese encephalitis was documented in 1871 in Japan. Approximately 4,000 people died during the 1924 epidemic in Japan, and nearly 2500 deaths occurred in South Korea in 1949. More than 3700 equids died during an epidemic in Japan in 1949 [170]. JE was detected in India, in 1955 and was confined to Tamil Nadu. Japanese encephalitis has emerged as a major problem and several outbreaks of Japanese encephalitis are reported from different parts of the country every year. The virus has invaded 21 rice-growing states of India [171, 172]. Deaths have been reported continuously from many states of India ranging from 466 in 2002 to 680 in 1999. Till 2007, 103,389 cases have been reported in India, with 33,729 deaths [173]. There had been 116 deaths in Malkangiri district of Orissa in India in 2016. There is no treatment for Japanese encephalitis. Vaccines are available for horses, swine and humans for prophylaxis. Vaccination has reduced the number of clinical cases among horses in endemic areas.

### 1.8 Rabies

Rabies is caused by the rabies virus, a neurotropic virus in the genus *Lyssavirus*, family *Rhabdoviridae.* The disease is important being zoonotic and highly fatal. All mammals are susceptible, but some species like dogs, jackals, coyotes, wolves, foxes, skunks, mongooses, and raccoons and bats act as reservoir hosts. Rabies cases have been reported across the globe in more than 150 countries. The worldwide cases of human rabies are estimated to be 55,000 or more every year with annual human mortalities of about 31,000 and 24,000 in Asia and Africa, respectively [174, 175]. A Rabies virus is most commonly transmitted through contact of saliva with damaged skin or mucous membranes, so strict barrier precautions should be used in all suspected cases.

Rabies is relatively rare in horses and usually less than 100 cases are reported in the United States every year [176, 177]. A large number of rabies cases have been reported in donkeys in Sudan over a period of ten years from 1992-2002 [17]. The clinical signs in horses are variable. The paralytic and dumb forms are most common, whereas the furious form is not as common as in other species. Clinical signs in the initial stages include ataxia and paresis of the hindquarters, lameness, recumbency, pharyngeal paralysis, and colic. The major clinical signs observed over the period include recumbency, hyperesthesia, loss of tail and anal sphincter tone, fever, ataxia and paresis of the hindquarters [178]. Affected animals usually die of cardio-respiratory failure within 2 to 5 days of onset of clinical signs; however, progression can be slower (up to 2 wk) in some cases. The incubation period for rabies is typically 1–3 months but may vary from 1 week to 1 year. The clinical signs in man may include malaise, fever or headache, as well as discomfort, pain, pruritis or other sensory alterations at the site of virus entry in early stages. Either furious form with hyper-excitability or a paralytic form characterized by generalized paralysis may predominate. Death usually occurs within 2 to 10 days. Survival is rare in clinically affected patients. Severe neurological disorders may occur as sequel to the disease in survivors.

Exposure to rabies is less common in people handling equines compared to small animals or wildlife. Vaccines are available for prevention of the disease in horses but there has been documented evidence of illness even in vaccinated horses [178]. For prevention pre-exposure vaccination is recommended for people in frequent contact with animals as they are more likely to be exposed to rabies.

### 1.9 Equine Influenza

Equine influenza is historically not known to affect humans but many scientists have mixed opinions. Xie and co-workers [179] have reviewed English, Chinese, and Mongolian scientific literature regarding evidence for equine influenza virus infections in man. On the basis of 16 publications, the authors could find considerable experimental and observational evidence that H3N8 equine influenza viruses have occasionally infected man. Morens and Taubenberger [180] found that from 1658 to the early 20th century, EIV outbreaks in horses often preceded 3 weeks or so human influenza-like-illnesses. The likely cause of human pandemic in 1889 has also been considered to be a H3N8 EIV [181-183]. Serological studies of the people who lived from 1892 era, have also demonstrated elevated antibodies against H3N8 EIV [184, 185]. Marois and colleagues [186] reported that viral culture and serological evidence to show that the virus came from humans and also speculated EIV outbreaks in horses may pose risk to humans.

## CONCLUSION

Equine viral diseases though restricted to certain geographical areas have huge impact on equine and human health. Many of these diseases have complex life cycles and require vectors for spread, while others can be contracted directly through aerosols. In some cases silent carriers like bats, pigs and passerine birds can contribute towards amplification of organisms and their spread to newer territories. A number of zoonotic diseases like West Nile fever, Hendra, VS, VEE, EEE, JE, Rabies have the potential for spread and ability to cause disease in human. Equine influenza is historically not known to affect humans but some scientists could find considerable experimental and observational evidence where H3N8 influenza virus has infected man. Despite our pursuit of understanding the complexity of the vector-host-pathogen mediating disease transmission, it is not possible to make generalized predictions concerning the degree of impact of disease emergence. The complex character and non-straightway behaviour of the human-natural systems in which host-pathogen systems are nested make specific prevalence of disease emergence or epidemics inherently difficult to predict. A targeted, multidisciplinary effort is required to understand the risk factors for zoonosis and apply the interventions necessary to control it.

## Figures and Tables

**Fig. (1):**
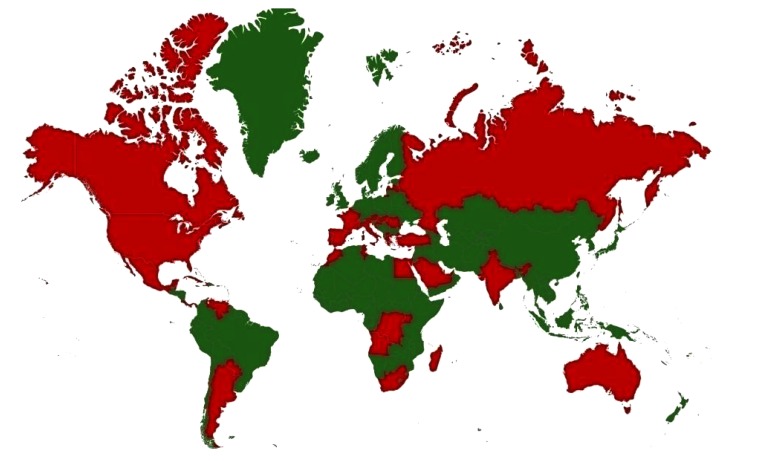
Disease distribution map of West Nile Fever showing worldwide incidence of the disease; Countries with history of infections/serological evidence are marked red and with no history are marked green.

**Fig. (2):**
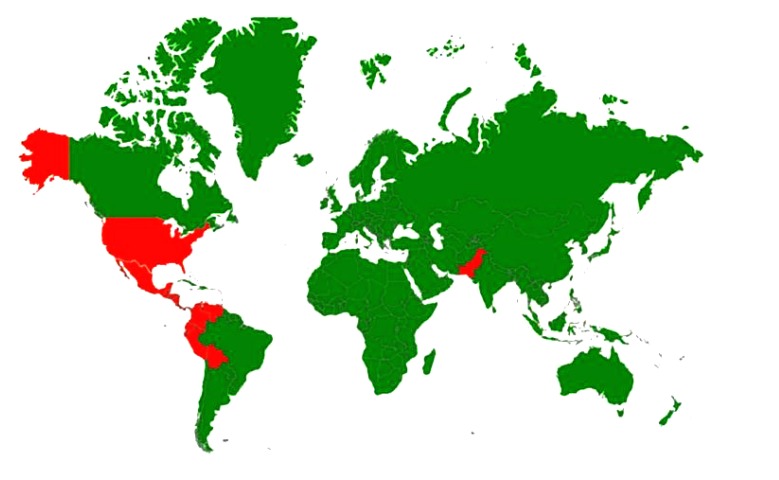
Disease distribution map of Vesicular Stomatitis showing worldwide incidence of the disease; Countries with history of infections/serological evidence are marked red.

**Fig. (3):**
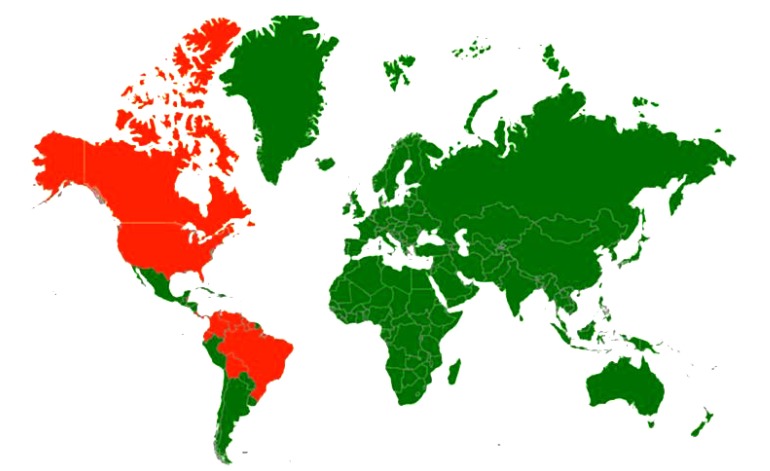
Disease distribution map of Eastern Equine Encephalitis showing incidence of the disease; Countries with history of infections/serological evidence are marked red.

**Fig. (4):**
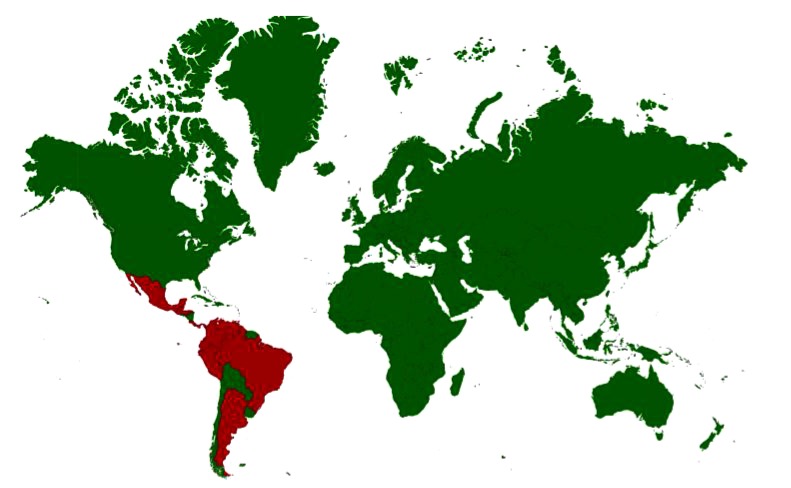
Disease distribution map of Venezuelan Equine Encephalitis showing incidence of the disease; Countries with history of infections/serological evidence are marked red.

**Fig. (5):**
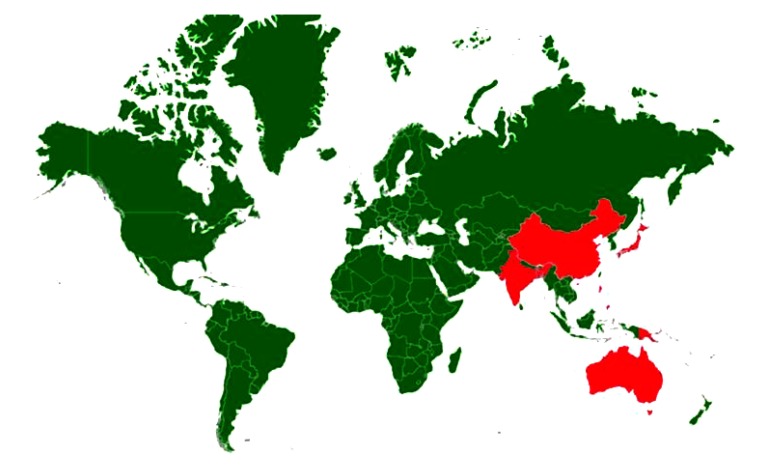
Disease distribution map of Japanese Encephalitis showing incidence of the disease; Countries with history of infections/serological evidence are marked red.
